# Mitochondrial protein translocation machinery: From TOM structural biogenesis to functional regulation

**DOI:** 10.1016/j.jbc.2022.101870

**Published:** 2022-03-26

**Authors:** Ulfat Mohd. Hanif Sayyed, Radhakrishnan Mahalakshmi

**Affiliations:** Molecular Biophysics Laboratory, Indian Institute of Science Education and Research, Bhopal, India

**Keywords:** Tom40, mitochondrial outer membrane, protein import pathways, TOM complex, transmembrane β-barrels, neurodegeneration, Bap31, B-cell receptor-associated protein 31, Hsp40/70/90, heat shock protein 40/70/90, *Hs*TOM, *Homo sapiens* TOM, IMM, inner mitochondrial membrane, IMS, intermembrane space, Mdm10, mitochondrial distribution and morphology protein, MIA, mitochondrial intermembrane space assembly machinery, MIM, mitochondrial import machinery, mOMP, mitochondrial outer membrane proteins, *Nc*TOM, *Neurospora crassa* TOM, OMM, outer mitochondrial membrane, SAM, sorting and assembly machinery, *Sc*TOM, *Saccharomyces cerevisiae* TOM, TIM, translocase of the inner mitochondrial membrane, TOM, translocase of the outer mitochondrial membrane, TOM–CC, TOM core complex

## Abstract

The human mitochondrial outer membrane is biophysically unique as it is the only membrane possessing transmembrane β-barrel proteins (mitochondrial outer membrane proteins, mOMPs) in the cell. The most vital of the three mOMPs is the core protein of the translocase of the outer mitochondrial membrane (TOM) complex. Identified first as MOM38 in *Neurospora* in 1990, the structure of Tom40, the core 19-stranded β-barrel translocation channel, was solved in 2017, after nearly three decades. Remarkably, the past four years have witnessed an exponential increase in structural and functional studies of yeast and human TOM complexes. In addition to being conserved across all eukaryotes, the TOM complex is the sole ATP-independent import machinery for nearly all of the ∼1000 to 1500 known mitochondrial proteins. Recent cryo-EM structures have provided detailed insight into both possible assembly mechanisms of the TOM core complex and organizational dynamics of the import machinery and now reveal novel regulatory interplay with other mOMPs. Functional characterization of the TOM complex using biochemical and structural approaches has also revealed mechanisms for substrate recognition and at least five defined import pathways for precursor proteins. In this review, we discuss the discovery, recently solved structures, molecular function, and regulation of the TOM complex and its constituents, along with the implications these advances have for alleviating human diseases.

Endocellular symbiotic evolution of mitochondria from proteobacteria led to well-established metabolic alterations in the cell, while also modifying the genome of the aerobic prokaryote (1, 2). Mitochondria contain >20% of the total cellular protein content, and yet mitochondrial DNA encodes only eight or 13 proteins in yeast and human, respectively. The vast majority of the ∼1000 to 1500 proteins (from yeast to humans) of modern mitochondria are nuclear-encoded and translated by cytosolic ribosomes (2, 3, 4, 5). Translocation of these proteins to their destination mitochondrial subcompartments is vital for mitochondrial biogenesis, biostasis, and bioenergetics (3, 4, 5, 6, 7). Not surprisingly, therefore, mitochondrial evolution was concurrent with the emergence of protein import machinery in the outer mitochondrial membrane (OMM).

The process of mitochondrial protein import is synchronized, and the import machinery is highly selective in recognizing and sorting widely different preprotein types (2, 4, 8, 9, 10). Two different import machineries have so far been identified, which are the translocase of the outer mitochondrial membrane (TOM) complex and the mitochondrial import machinery (MIM) (in yeast) (11, 12). After its synthesis in the cytosol, each polypeptide targeted for mitochondrial import is maintained in its transport-competent state by the cytosolic chaperones (holdases) that also prevent their aggregation (13, 14). These chaperones (*e.g.*, Hsp40, Hsp70 (15, 16)) additionally facilitate the targeting of the nascent polypeptide to the OMM, wherein the import machinery target these proteins to the OMM or mitochondrial intermembrane space (IMS). The heteromolecular TOM complex is the primary entry gate for >90% of mitochondrial proteins, with different subunits of this complex playing distinct roles in preprotein recognition and import (2, 9, 17, 18, 19, 20, 21, 22).

Our current knowledge of the TOM complex structure, organization, and function, is derived largely from studies in *Saccharomyces cerevisiae* (*Sc*TOM) and *Neurospora crassa* (*Nc*TOM), with work from human TOM (*Hs*TOM) being more recent. This review describes the evolution, structure, and dynamics of the TOM complex, outlines regulatory roles and communication networks identified for specific TOM complex subunits, as identified in fungal mitochondria. We also discuss the consequences of misregulation, which can result in disease states in humans.

## Structural characteristics of the TOM complex

**Figure 1:**
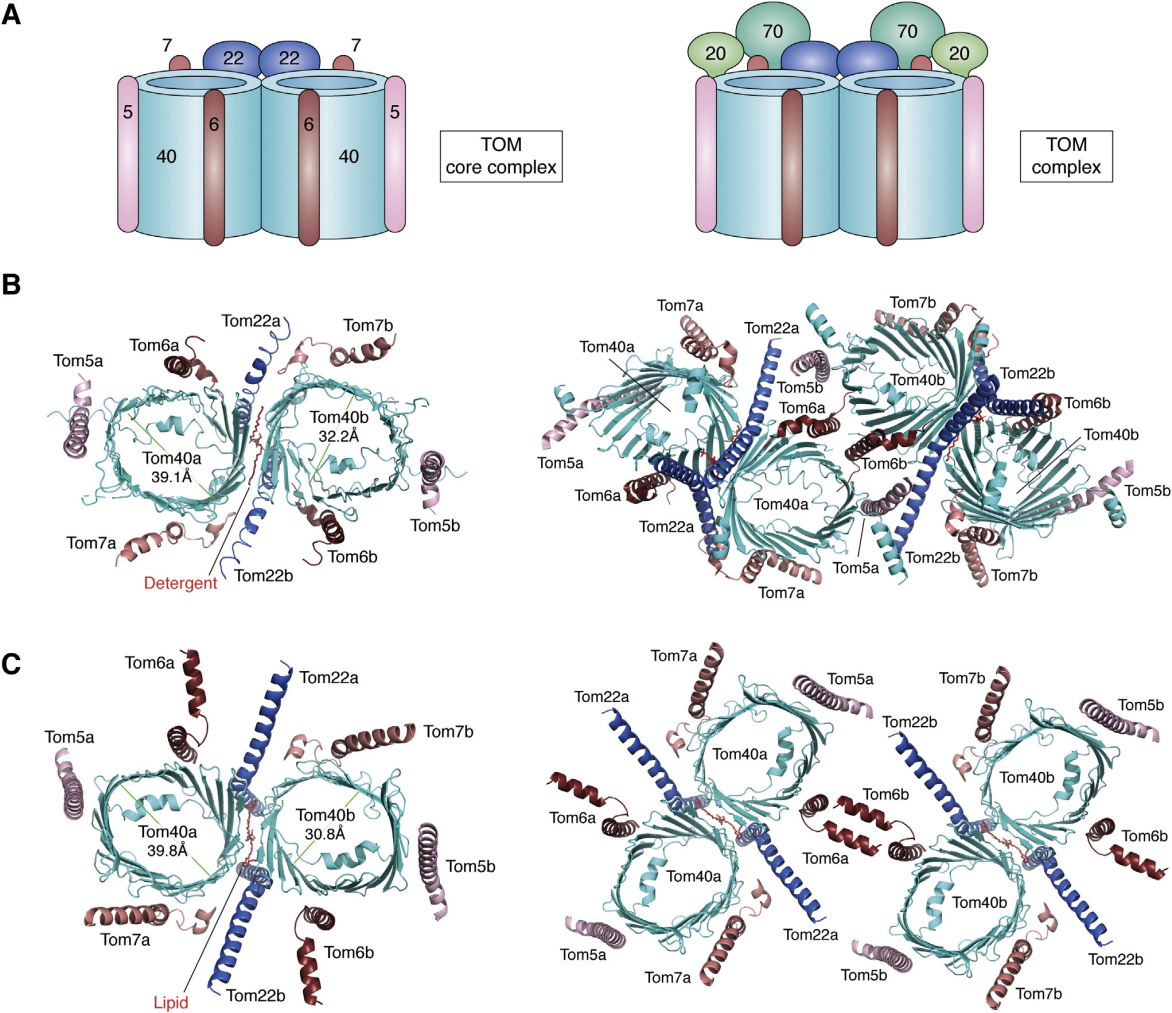
**Structural organization of the TOM–CC.***A*, components of the TOM–CC and TOM complex (additionally containing one or two copies each of the Tom20 and Tom70 receptors) are shown as *cartoon* representations. *B* and *C*, *ribbon* diagrams of the *Saccharomyces* (*B*) (PDB ID: 6UCU, 6UCV) and human (*C*) (PDB ID: 7CK6) TOM–CC structures deduced with cryo-EM (26, 27, 28, 29). Pore diameters at the longest and shortest edges are shown in Tom40a and Tom40b, respectively. Both dimeric and tetrameric forms of the TOM–CC have been reported. Tetrameric TOM–CC in (*C*) was generated using coordinates for the dimer. Tom40 dimerization is facilitated by anchoring interactions of the Tom22 helix and sandwiched detergent/lipid molecules. Tom5, Tom6, and Tom7 associate along the three other faces of Tom40. Tetramerization of the TOM–CC in both *Sc*TOM (*B*) and *Hs*TOM (*C*) occurs through Tom6 and stabilized by Tom5–Tom22 interaction in *Sc*TOM. Note how similarities in structure and organization are conserved in both *Sc*TOM and *Hs*TOM. *Hs*TOM, *Homo sapiens* TOM; *Sc*TOM, *Saccharomyces cerevisiae* TOM; TOM, translocase of the outer mitochondrial membrane; TOM–CC, TOM core complex.

Despite its importance for cell viability, constituents of the TOM complex was first identified only in 1989 (23, 24), while its structure and organization remained elusive for nearly 3 decades. Cryo-EM has been a powerful tool in revealing the structures of *Nc*TOM complex in 2017 (25), *Sc*TOM in 2019 (26, 27), and *Hs*TOM in 2020 (28) and 2021 (29). These structures revealed that the completely assembled TOM complex consists of seven nuclear-encoded subunits: the central channel Tom40, three small subunits Tom5, Tom6, and Tom7, and the three receptors Tom20, Tom22, and Tom70 (Fig. 1*A*). The Tom40 channel, small Tom subunits, and Tom22 form the TOM core complex (TOM–CC) (Fig. 1*A*, left), which binds the regulatory components Tom20 and Tom70 to form the TOM complex (Fig. 1*A*, right) (2, 30, 31). In human mitochondria, TOMM40, TOMM5, TOMM6, TOMM7, TOMM22, TOMM20, and TOMM70A are the components of the TOM complex (28). Structural organization of the TOM–CC is conserved evolutionarily across all three organisms. Tom40 forms the principal protein-conducting pore for import of nuclear-encoded precursor proteins across the OMM in an ATP-independent manner (17, 20, 32, 33, 34). Tom5, Tom6, and Tom7, along with Tom22, are essential for both the assembly and stability of the TOM complex (25, 26, 27, 28, 30, 35, 36, 37, 38, 39, 40, 41, 42). Tom20 and Tom70 associate dynamically with the TOM complex and confer specificity in both substrate selection and import (2, 43, 44, 45, 46).

### Core Tom40 structure

Tom40 is evolutionarily distinct with no bacterial homolog. Cys-scanning and protease accessibility studies showed that Tom40 forms a 19-stranded β-barrel with barrel closure through parallel hydrogen bonds as seen in porins (2, 8, 9, 25, 26, 27, 28, 29, 46, 47, 48, 49). In *S. cerevisiae*, three dynamic extramembranous α-helical segments are present (26, 27). The two N-terminal helices transit the β-barrel interior from the cytosolic side to the IMS, with α1 residing on the IMS side and α2 spanning the pore interior. The C-terminal α3 is directed from the IMS into the barrel. Surface electrostatic calculations show that the β-barrel lumen is highly negatively charged, which attracts positively charged polypeptides for translocation (26, 27).

Tom40 forms a modestly elliptical pore of ∼40 Å diameter at its longest point (Fig. 1, *B* and *C*, left), which can accommodate two incoming α-helices at a time (18, 25, 26, 27, 28, 29). The transmembrane span of the barrel (loop-to-loop distance) is ∼90 Å, with a hydrophobic span of ∼30 Å. The stable conformation of Tom40 is either a dimer or tetramer (Fig. 1, *B* and *C*) (25, 26, 27, 28), although a trimeric state has additionally been proposed for *Hs*Tom40 (29). In its dimeric conformation, the inner cross-sectional diameter of the two elliptical pores is about 40 Å by 30 Å, excluding the N-terminal α-helices (dimeric *Nc*TOM–CC has an average pore diameter of 22 Å (25)). The outer surface of Tom40 possesses a positive potential on the IMS side along the dimer interface, with the pore lining near the cytosolic side possessing a negatively charged region (25, 26, 27, 28). The two Tom40 subunits do not share a large interaction surface, and instead, two Tom22 receptors bind and stabilize the dimeric Tom40 pores (25, 26, 27, 28, 30). Interestingly, a tetrameric structure formed by lateral stacking was also detected, and is the likely result of dimerization of the dimer (26, 27), and connected by two Tom6 subunits (26, 27, 28). A trimeric form has been reported in both yeast (27, 50) and humans (29). Higher oligomeric structures of Tom40 have not yet been identified, and it is thought that the stable form of this protein is a dimer (18, 21, 47, 49).

### Structural organization of Tom40 with other TOM–CC subunits

Cryo-EM structures show that dimeric Tom40 is stabilized by two copies each of Tom22, Tom5, Tom6, and Tom7 and form the TOM–CC (25, 26, 27). The single-pass transmembrane helices Tom5, Tom6, and Tom7 (TOMM5/6/7 in *Hs*TOM–CC (28, 29)) bind peripherally at distinct sites around each Tom40 pore and function in TOM-CC stability and assembly (35, 36, 38, 40, 51, 52, 53, 54, 55, 56). Structural studies (25, 26, 27, 28, 29, 49) show that Tom5 interacts with Tom40 at strands β10 and β11 at the cytosolic site, Tom6 at β13–β15, and Tom7 at β3–β6. Tom5 is a long α-helix, with its C-terminal half embedded in the membrane and its cytosolic negatively charged N-terminal domain interacting with the N-terminus of Tom40 and the incoming polypeptide (51). The kinked helical structure of Tom6 bears the transmembrane segment at its C-terminus, while the partly helical N-terminal segment is located in the IMS (25). Tom6 additionally interacts directly with Tom22 and stabilizes the Tom40–Tom22 interaction at the dimer interface.

Tom7 is a Z-shaped kinked helix (25) and sits in contact with Tom40 through its central transmembrane domain. The C-terminal hook spans the IMS leaflet of the OMM (26, 27), forming several conserved polar interactions near the membrane boundaries, which together play a crucial role in subunit specificity and affinity (26, 27). Studies indicate that Tom6 and Tom7 are functional antagonists of each other in lower eukaryotes (40). Tom6 deletion lowers its stabilizing effect on the Tom6–Tom40–Tom22 complex (38, 57). In contrast, yeast Tom7 has a destabilizing role (37, 40, 58). The presence of Tom7 in yeast causes dissociation of Tom20–Tom22 from Tom40 and partial dissociation of Tom20 and Tom22 (57, 58). Unlike *Nc*TOM (36, 53) and *Sc*TOM (40, 58), both TOMM6 and TOMM7 appear to have stabilizing roles in the human TOM complex (28). Knockdown of human TOMM7 increases tetrameric Tom40 levels (28, 29, 59).

Tom22 is the Tom40–CC associated central preprotein receptor, with a single transmembrane α-helical anchor and two extramembranous hydrophilic domains (41, 60). In the *Sc*TOM dimer, the two Tom40 subunits form direct interactions *via* hydrophobic side chains of β1-β19-β18 toward its cytosolic side, while two Tom22 subunits are wedged between the Tom40 barrels on its IMS side (26, 27). The cytosolic N-terminal subunit is the hydrophilic multifunctional receptor for preproteins (30, 42, 61, 62, 63). It recognizes the signal sequence to be imported through Tom40 and also serves as docking sites for Tom20 and Tom70 (25, 26, 27, 29). The IMS-exposed C-terminal domain of Tom22 is crucial both for TOM complex stability, as well as to receive the imported preprotein and conduct it to its respective machinery (30, 61), particularly the translocase of the inner mitochondrial (TIM)23 complex *via* interactions formed with Tim21 and Tim50 (64, 65, 66, 67, 68). This domain additionally induces release of the precursor protein from Tom40. Additional roles of these TOM–CC subunits remain to be deduced.

### Structural organization of the TOM complex

Tom20 and Tom70 are the major Tom40-associated receptors, which bind to the TOM–CC to form the TOM complex (43, 44, 45, 46, 69, 70, 71, 72, 73). They are anchored to the OMM by a single transmembrane α-helix, and both proteins possess a hydrophilic segment oriented cytosolically (45). Tom20 is anchored in the OMM by its N-terminal α-helix. The cytosolic C-terminal segment, which functions as the receptor, is an α-helix–rich structure with a hydrophobic groove that accommodates the presequence region of the incoming polypeptide (44, 71). Tom70 contains an N-terminal hydrophobic α-helix anchor. The yeast Tom70 crystal structure revealed that the cytosolic domain of Tom70 is a bundle of 26 α-helices (A1–A26), and most of them are arranged in a tetratricopeptide repeat motif. The monomer of Tom70 forms a suprahelical structure organized through its N-terminal (A1–A7) and C-terminal (A8–A26) helices (72). In yeast, the Tom22 cytosolic domain provides docking sites for Tom20 and Tom70 (26, 27, 30, 42, 74).

As seen with the TOM–CC, the TOM complexes also appear to share similarities and differences between yeast and human mitochondria. Cytosolic heat shock proteins Hsp70 (yeast) and the multi-chaperone complex of Hsp70 and Hsp90 (human) deliver preproteins to Tom70 and TOMM70A, respectively (15, 16, 73, 75, 76). Tom20 (TOMM20 in humans) acts as the receptor for precursor proteins carrying an N-terminal signal sequence, and Tom70 (TOMM70A in humans) for proteins which carry an internal targeting sequence (3, 4, 77). Interestingly, TOMM20 assists TOMM70A in translocating preproteins to TOMM40 in *Hs*TOM (45), while in *Sc*TOM, Tom22 acts as the receptor for channeling preproteins to Tom70 (2, 74). Further structural and functional studies of TOM complex regulators are required to identify mechanistic insights on the evolution of these systems and their molecular differences across eukaryotic mitochondria.

## TOM complex biogenesis

**Figure 2:**
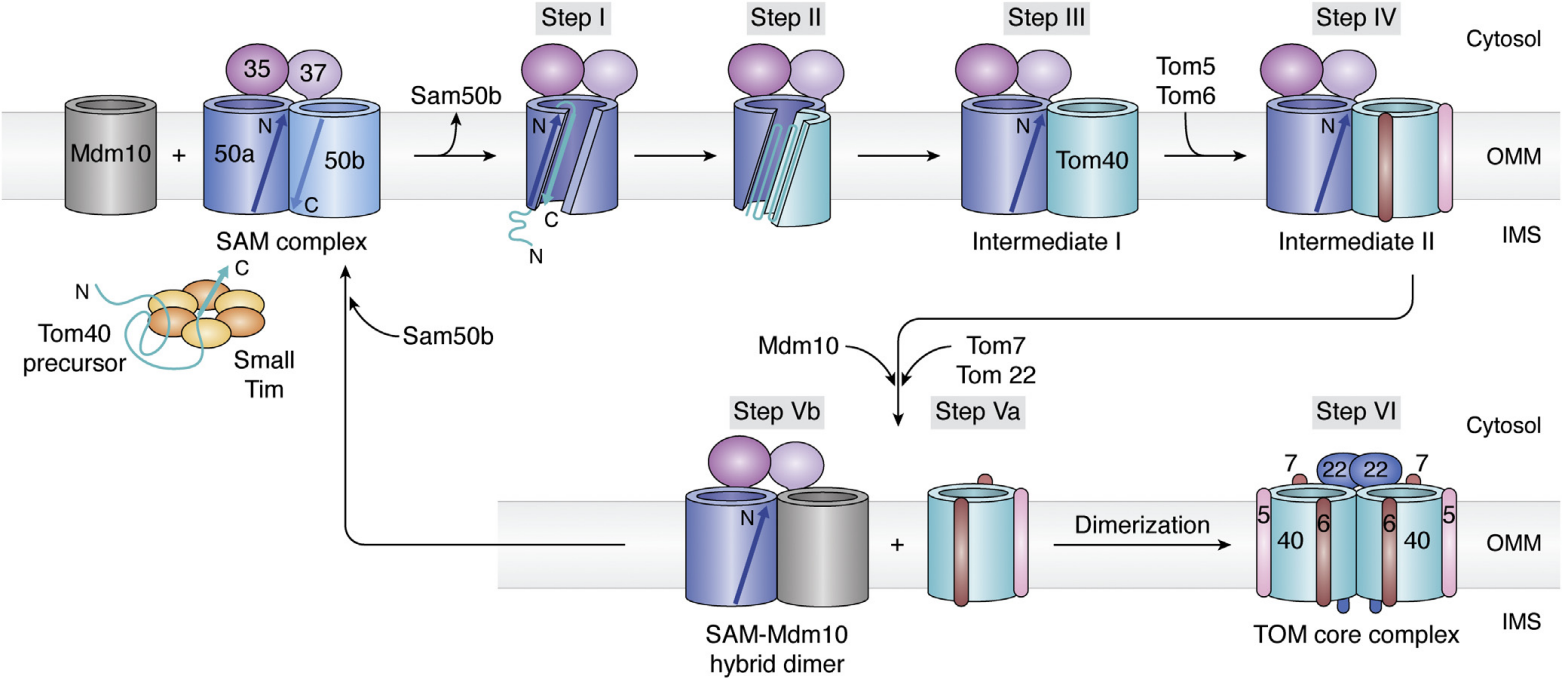
**β-Barrel switching mechanism for SAM-catalyzed assembly of the yeast TOM–CC.** Recent cryo-EM structures (56) of the assembly intermediates support a β-barrel switch mechanism for SAM-mediated Tom40 folding. The SAM complex (comprised of Sam35-bound Sam50a and Sam37-bound Sam50b) serves as the chaperone complex for an incoming nascent β-barrel polypeptide. Interaction of the small Tim holdases carrying the nascent Tom40 precursor with the IMS face of Sam50a triggers the dissociation of Sam50b (step I), which initiates the folding of Tom40 in the membrane (step II), leading to formation of the SAM–Tom40 hybrid barrel (Intermediate I) (step III). Sam37 stabilizes the membrane-inserted Tom40 β-barrel by binding at its cytosolic face. The association of Tom5 and Tom6 to the hybrid barrel results in the formation of Intermediate II (step IV). Next, the binding of Tom7 and Tom22 (step V) triggers the dissociation of Tom40/5/6 and its release into the membrane (step Va). The subsequent dimerization of Tom40/5/6/7/22 gives rise to the TOM–CC (step VI). Mdm10 assists Tom40 release, by associating with Sam50a (step Vb). Binding of Sam50b to the hybrid Mdm10–Sam50a/35/37 complex allows reformation of the SAM complex, restoring the chaperone function of the SAM complex (8, 80). The cryo-EM structures (56) also reveal how Tom7 facilitates the dissociation of Tom40/5/6 from the SAM complex (see Fig. 3). Mdm, mitochondrial distribution and morphology; SAM, sorting and assembly machinery; TOM, translocase of the outer mitochondrial membrane; TOM–CC, TOM core complex; IMS, intermembrane space.

One of the earliest studies to characterize the assembly of the core TOM complex (then referred to as the general import pore) was in yeast between 1998 and 2001 (57, 78). While several folding and assembly mechanisms have been proposed, studies from pull-down experiments (57) to cryo-EM (56) have together provided a generalized assembly process for the TOM complex that is largely method-independent. The OMM sorting and assembly machinery (SAM; Sam50 is the core β-barrel of SAM complex) is vital for the insertion and folding of Tom40 (9, 56, 79, 80) and other α-helical constituents, namely Tom5, Tom6, Tom7, and Tom22 (81). Various molecular mechanisms for the Sam50-assisted folding have been proposed (discussed in (79, 80, 82, 83)). The most recent studies in yeast show that the process occurs through a β-barrel switching model (identified earlier for SAM-mediated assembly of nascent Sam50 (80)) in yeast (Fig. 2) (56).

The stepwise event starts with the recognition of the Tom40 precursor by the SAM complex (56). Here, the SAM complex comprises of two Sam50 subunits, Sam50a and Sam50b, and one subunit each of Sam35 and Sam37 bound at the cytosolic face to Sam50a and Sam50b, respectively (80). The incoming nascent Tom40 precursor triggers the displacement of Sam50b, and the resultant insertion of Tom40 in the membrane leads to the formation of the SAM–Tom40 hybrid (Intermediate I) comprised of one subunit each of Sam50a, Sam35, Sam37, and Tom40 (Fig. 2). The negatively charged α-helices 6 and 7 and the positively charged α-helix 8 of Sam37 interact with the polar lumen of Tom40 and stabilize the SAM–Tom40 hybrid complex.

The SAM complex facilitates integration of the Tom40–Tom5 intermediate, through the formation of a large SAM–Tom40/5 assembly (39, 83). This SAM–Tom40/5 assembly is the binding-competent for Tom6, which independently undergoes membrane insertion in a Mim1-dependent manner (Mim1 is a component of MIM). Indeed, Mim1 is involved in the integration of other α-helices of the TOM complex (12, 39, 84, 85). Mim1- or Tom5-deficient mitochondria accumulate Tom40 in the first stage of TOM assembly, and it has been proposed that Tom5 promotes the progression of the assembly to the second stage (39). The hybrid SAM–Tom40/5/6 is Intermediate II (Fig. 2).

**Figure 3:**
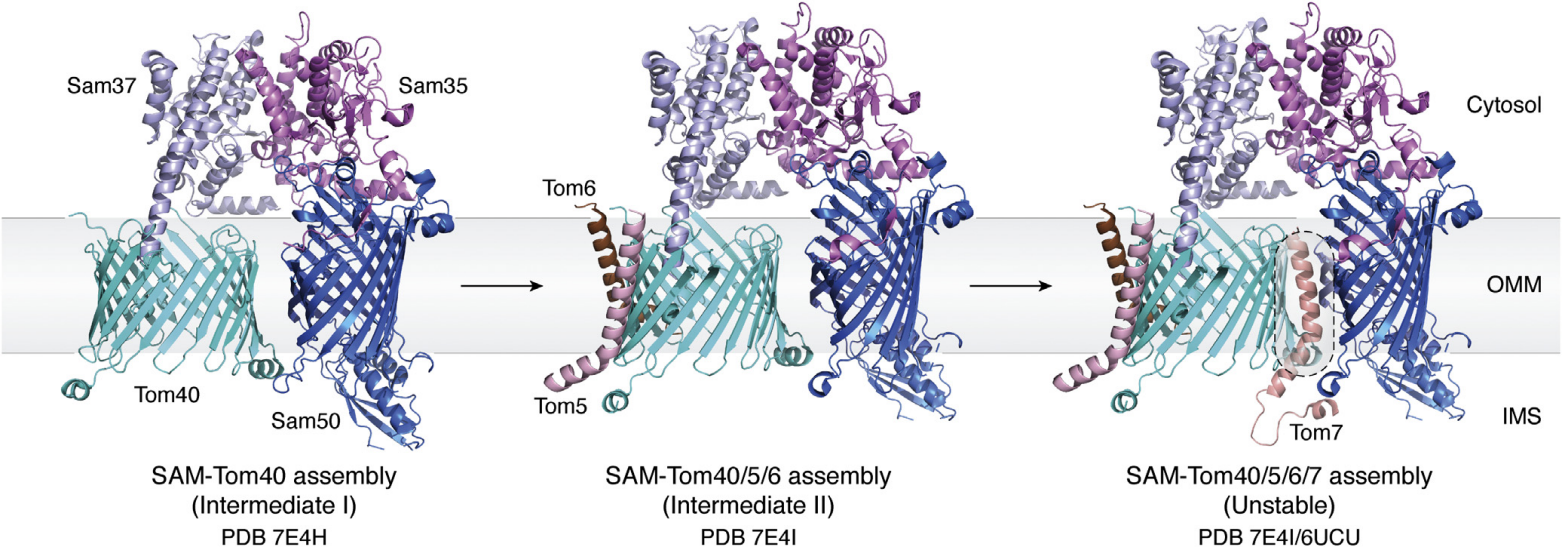
**Formation of TOM–CC from the SAM–Tom40 hybrid barrel.** Tom40 of the SAM–Tom40 hybrid barrel (*left*; Intermediate I; PDB ID: 7E4H) associates directly with and binds Tom5 and Tom6 through electrostatic interactions, giving rise to Intermediate II (*middle*; SAM–Tom40/5/6; PDB ID: 7E4I). Tom5/6 binding additionally stabilizes Tom40, while Sam35 stabilizes the elliptical Sam50 barrel during this process (56). Cryo-EM structures of the Tom40–SAM complex reveal a common binding site for both Sam50 and Tom7 on the Tom40 β-barrel (*right*; *purple oval*; Tom7 coordinates from PDB ID: 6UCU superimposed on PDB ID: 7E4I), indicating that formation of Tom40–Sam50 and Tom40–Tom7 structures is mutually exclusive. Therefore, the association of Tom7 is anticipated to trigger the release of Tom40/5/6 from the SAM–Tom40 hybrid assembly. Tom7 therefore plays a vital role in the dissociation of Intermediate II (56). SAM, sorting and assembly machinery; TOM, translocase of the outer mitochondrial membrane; TOM–CC, TOM core complex.

The formation of the mature TOM complex is preceded and stimulated by the SAM–Tom40/5/6 association. Tom7 shares the same binding face on the Tom40 barrel as Sam50 (Fig. 3). Hence, the association of Tom7 with Intermediate II triggers the release of Tom40. Sam50 also heterodimerizes with Mdm10 (mitochondrial distribution and morphology protein; 19-stranded β-barrel; (86)). This SAM–Mdm10 complex is essential for the membrane integration of Tom22 and simultaneous release of Tom40/5/6/7 from Intermediate II (58, 87). Tom22-mediated dimerization of Tom40 results in formation of the TOM–CC. Other oligomeric states of the TOM complex (trimeric (27, 29, 49, 50) and tetrameric (26, 28) forms) are likely assembled after the TOM–CC is formed.

In yeast, Tom7 has an antagonistic role in the maturation of the complex (40). Tom7 inhibits TOM complex biogenesis by promoting the premature release of Tom22 (57), while affecting the interaction of Tom5 and Tom6 with the SAM–Tom40 complex (40). Tom7 also directly affects Tom22 biogenesis (40), wherein Tom7 binds Mdm10 through its transmembrane segment (58) and promotes dissociation of the SAM–Mdm10 complex essential for Tom22 biogenesis. Tom7 therefore acts as a regulatory clock by delaying the biogenesis of the two topologically distinct Tom40 and Tom22 subunits of the TOM complex (40). The interaction surface of Tom7 with both Mdm10 and Tom40 is believed to be similar (58), and a timed release of Tom40 from the SAM complex by recruitment of Mdm10 is executed by this subunit. Whether the general mechanism of TOM assembly characterized in yeast is also conserved in humans remains to be established.

## Mitochondrial preprotein import

**Figure 4:**
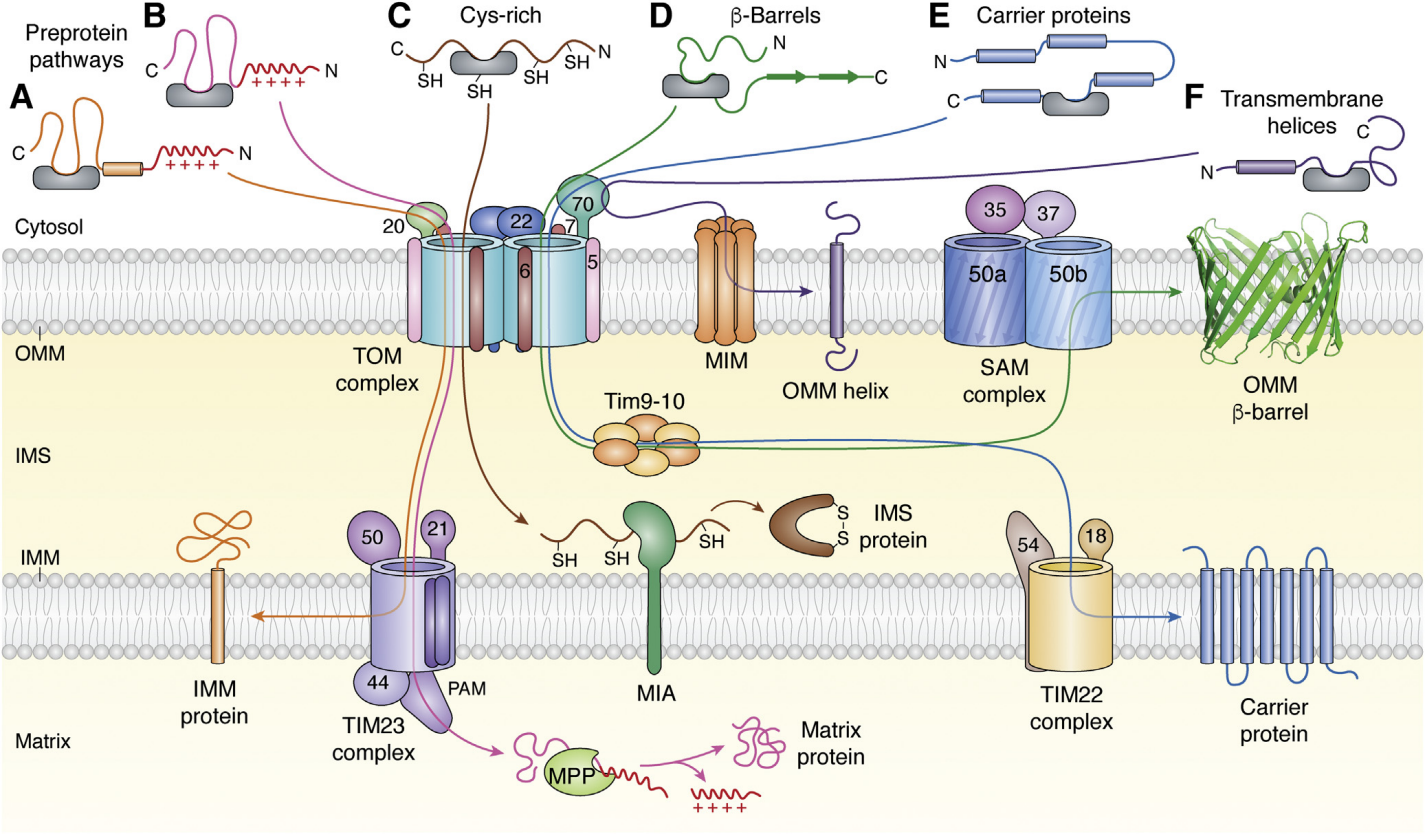
**Targeting signal sequences for preprotein import by the TOM complex.** Proteins imported across the OMM, with various mitochondrial subcompartments as their destination, possess cleavable or noncleavable sequences and can be charged or hydrophobic. The import is orchestrated by a specific TOM complex receptor, and the molecular pathway used by the preprotein within the Tom40 channel is dictated by the polarity of the targeting sequence. Preprotein pathways (*A*; *orange*) and (*B*; *magenta*), destined for the IMM and matrix respectively, carry an N-terminal cleavable signal sequence, recognized by Tom20. Tom22 interacts with TIM23 of the IMM for coordinated preprotein handover between both OMM and IMM import machineries. TIM23 triggers lateral release of the processed polypeptide in the IMM (*A*) or completes the import into the matrix (*B*), after processing by mitochondrial processing peptidases (MPP). IMS proteins with a Cys-rich internal hydrophobic signal sequence (pathway *C*; *brown*) are imported in a Tom22-depleted dimeric Tom40 (50). The preprotein is handed over to the IMM-anchored MIA machinery, which catalyzes disulfide bond formation and release of these polypeptides in the IMS. (*D*; *green*) OMM β-barrels with a noncleavable C-terminal internal β-signal sequence are recognized first by Tom70 followed by Tom22, for import through Tom40. Tim9–Tim10 holdases handover the unfolded polypeptide to the SAM complex for assembly in the OMM. Carrier proteins (*E*; *blue*) are imported first through Tom70–Tom40, carried by small Tims to TIM22, for their import and release in the IMM. (*F*; *purple*) Transmembrane helices of the OMM with a noncleavable signal anchor sequence are imported by MIM directly into the OMM, with assistance only from Tom70. Figure inspired from (2, 9, 187). IMM, inner mitochondrial membrane; IMS, intermembrane space; MIA, mitochondrial intermembrane space assembly; MIM, mitochondrial import machinery; OMM, outer mitochondrial membrane; SAM, sorting and assembly machinery; TIM, translocase of the inner mitochondrial membrane; TOM, translocase of the outer mitochondrial membrane.

Cytosolically-synthesized mitochondrial proteins are imported successfully into one of the four distinct mitochondrial compartments: OMM, IMS, inner membrane (IMM), and matrix. While a few single-pass transmembrane helices are inserted directly in the OMM by the MIM (12), the vast majority of the mitochondrial proteins rely on the TOM complex (Fig. 4) (2, 3, 5, 6, 8, 9, 10, 27, 46, 77, 83, 88, 89, 90, 91, 92, 93, 94, 95, 96, 97). The TOM complex recognizes preproteins that carry a specific mitochondrial targeting signal, through receptor subunits on its cytosolic face (*cis* side), located on the extramembranous domains of Tom20 and Tom22 (2, 8, 9, 27, 45, 46, 73, 75, 96, 98). Tom40 acts as the core protein–conducting channel (17, 27) and may additionally play the role of an insertase while promoting the release of proteins into the OMM (99). While discrete TOM complexes may exist for the import of specific mitochondrial protein groups (63, 100), whether this can be generalized or may correspond only to the TOM–CC requires validation (2).

Information for localization of the preprotein to specific mitochondrial compartments is determined both by its primary sequence and by secondary structure properties (101). The signal sequence, which directs the nascent polypeptide–chaperone complex to TOM receptors, are of two categories: (i) cleavable, and (ii) noncleavable (internal) (Fig. 4). For example, tail-anchored proteins of the OMM are imported by both TOM and MIM (10, 90), while OMM β-barrels require the TOM complex. IMS-specific proteins with structural disulfide bonds use their cysteine-rich internal amphipathic helix for IMS targeting (97). Successful localization of proteins to the IMM and matrix requires functionally and kinetically coupled reactions of the TOM and TIM complexes (2, 3, 63, 68, 96, 102). Once the polypeptide traverses the OMM, a perfect temporal and spatial coupling of TOM–TIM23 complexes occurs sequentially for the import of preproteins with an N-terminal targeting signal and destined for IMM or matrix, and the TOM–TIM22 complex for carrier proteins of the IMM with an internal targeting sequence (2, 3, 5, 6, 8, 9, 62, 68, 96, 103). Here, we describe the various preprotein import mechanisms of the TOM complex.

### Substrate-dependent changes in the TOM complex

Although little is known about dynamic structural alterations in the TOM complex, how a preprotein induces changes in the TOM complex can be explained in two steps. Modification in the architecture at step one occurs when a preprotein reversibly binds to the TOM complex receptor (*cis* side) on the mitochondrial surface (44). This preprotein–receptor interaction is detected and relayed to Tom40. Tom40 pore opening is induced and preprotein import ensues, while the auxiliary TOM receptors enhance the efficacy of this translocation process (98, 104).

The second step of conformational changes arises during and after the precursor protein is imported across the OMM (98, 104, 105). Once the preprotein is in the IMS (*trans* side), changes occur in the Tom40 chemical environment due to binding of both the preprotein and mature regions of the polypeptide being imported with the Tom40 channel interior (98, 104, 105). It is thought that this change is mainly triggered by the preprotein’s N-terminal extension. In the IMS, both the presequence and mature regions are in close contact with Tom40 (98, 104, 106), maintaining the preprotein in its translocation-competent state and preventing the polypeptide from aggregation until the handover to IMS chaperones is completed (97, 107).

Tom40, Tom5, Tom6, and Tom7 constitute the core translocation pore of the TOM complex. Tom5 mediates the transfer of preproteins from the Tom20–Tom22 receptor complex to the translocation pore of Tom40 (35, 51, 108). Overall conformational changes in and rearrangement of Tom40 (*e.g.*, dimer–trimer interconversion (50); discussed later) can be triggered by the targeting sequences of the preproteins and their translocation. Tom22 regulates the interconversion frequency between open and closed states of the TOM complex, triggering channel opening only in the presence of preproteins (30). Studies have also shown that mechanoregulation by lateral diffusion can switch the TOM–CC between open and closed states of one or both Tom40 channels (55). These alterations of Tom40 are expected to affect both the β-barrel structure and its interaction with other TOM proteins and can represent the two functional stages of the TOM complex (preprotein binding and *cis*–to–*trans* preprotein translocation). Tom6 and Tom7 play a vital role in influencing the stability of the TOM complex structures, during both stages of polypeptide translocation (96).

### TOM–TIM23 interaction for precursors with a presequence

**Figure 5:**
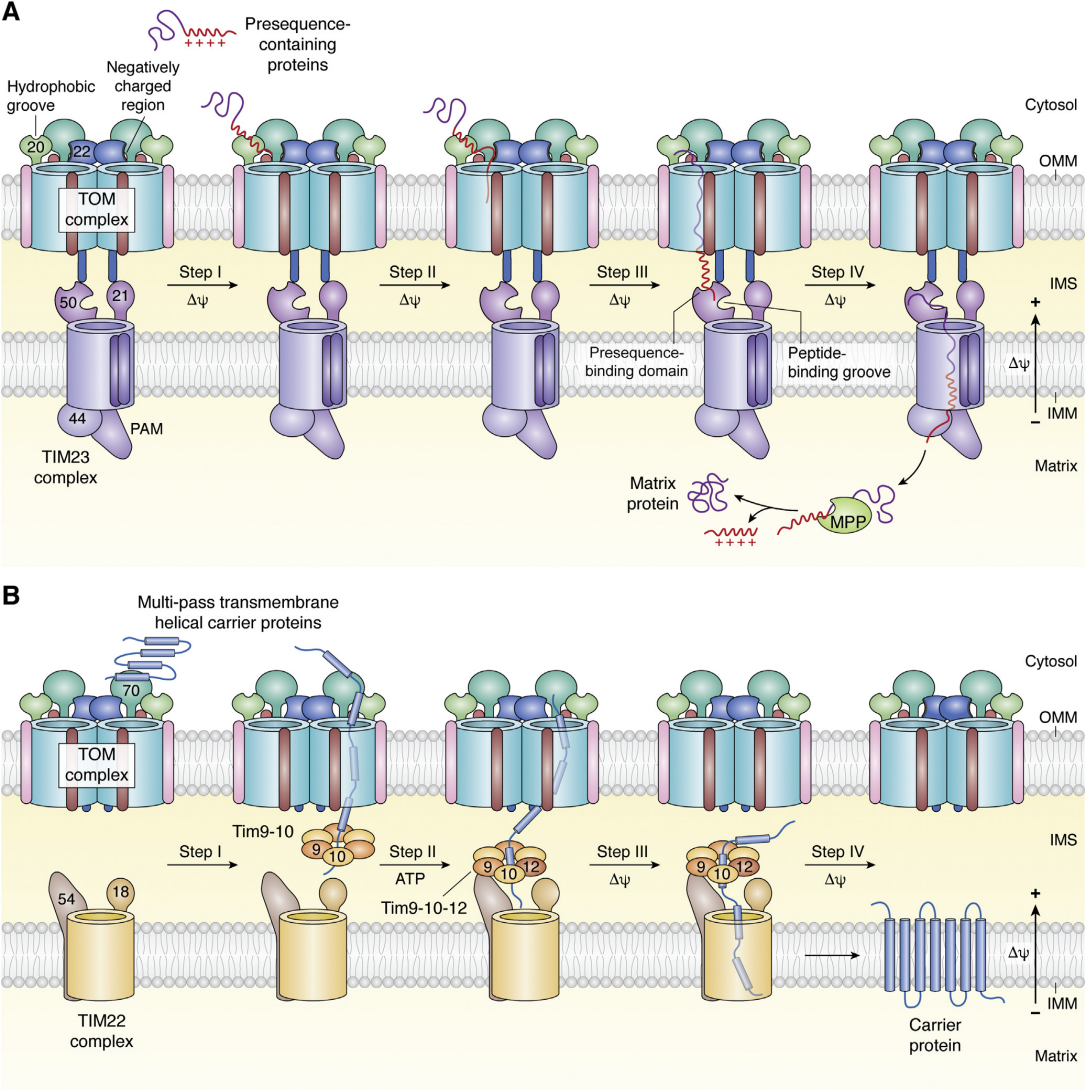
**TOM–TIM interaction for protein import.***A*, presequence-containing proteins targeted to the mitochondrial matrix require coordinated transfer from the TOM to the TIM23 complex. First, Tom20 recognizes the positively charged presequence and transfers it to Tom22 (step 1). The latter directs the polypeptide to the negatively charged lumen of Tom40 for transport across the OMM through favorable electrostatic interactions (step 2). At the IMS face, the presequence is recognized and bound by the extramembranous domain of Tim50, for transfer to the TIM23 complex (steps 3–4). MPP cleaves the presequence, releasing the mature folded protein in the mitochondrial matrix. The presequence handover from TOM to TIM23 is coordinated by Tom22 and Tim50 in the OMM and IMM, respectively. The matrix face of TIM23 also contains Tim44 and the ATP-dependent presequence translocase-associated motor (PAM). Figure inspired from (68). *B*, import of multipass transmembrane helical carrier proteins of the IMM is executed by the TIM22 complex. Tom70 recognizes the signal sequence and Tom40 imports it across the OMM (steps 1–2). At the IMS face, small Tim9–Tim10 chaperones capture and retain the polypeptide in its unfolded state (14). The hybrid Tim9–Tim10–Tim12 complex that is formed next, hands over the polypeptide to the TIM22 complex, which successfully inserts and folds the carrier protein into the IMM (steps 3–4). Figure inspired from (63). Both the TOM–TIM import processes are driven by the potential across the IMM and are ATP-independent. IMM, inner mitochondrial membrane; IMS, intermembrane space; MPP, mitochondrial processing peptidase; OMM, outer mitochondrial membrane; TIM, translocase of the inner mitochondrial membrane; TOM, translocase of the outer mitochondrial membrane.

The TIM23 complex is the most abundant import machinery of the IMM and is required for import of presequence proteins (Figs. 4 and 5*A*) (96, 109, 110, 111, 112). Tim23 is the major channel-forming protein (112, 113, 114). Tim23, Tim21, and Tim50 together establish a communication link between TOM and TIM23 complexes, *via* their IMS-exposed domains, while Tim14 facilitates this process (112, 115, 116, 117). The majority of proteins targeted to the IMM and matrix carry an N-terminal cleavable signal sequence (Fig. 5*A*), containing ∼8 to 100 positively charged residues that form an amphipathic α-helix (71, 96, 109, 112). The TIM23 receptors additionally transport hydrophobic preproteins with internal signal sequences (*e.g.*, metabolite carriers) (70, 111, 112, 114, 118).

Tom20 is the primary receptor for IMM and matrix proteins. As the precursor protein emerges from the Tom40 pore (111), Tim50 interacts with and stimulates binding of the preprotein to the *trans* (IMS) domain of Tom22 (Fig. 5*A*) (64, 66, 67, 68, 110, 112, 119). This establishes the interaction of Tim21 with Tom22 (64), catalyzing the simultaneous release of the presequence from the TOM complex and its interaction with Tim23 (64, 66, 112). The activated Tim23 pore now binds to the incoming polypeptide through its N-terminal hydrophilic domain. The membrane potential across the IMM promotes opening of the Tim23 channel and creates an electrophoretic effect that drives the transport of the presequence (112, 114, 120). This perfect presequence handing over by formation of the TOM–TIM23 supercomplex is activated by the precursor protein and is stabilized by Tom22 and Tim50 (Fig. 5*A*) (66, 67, 109, 111, 112, 113, 117). These polypeptides carry an additional internal targeting sequence, which retain them in an import-competent state. Once inside Tim23, the preprotein is released laterally and imported into the matrix (Fig. 5*A*) for further processing by mitochondrial processing peptidases (121). Proteins destined for the IMM carry this internal targeting signal sequence near their transmembrane domain(s), which is followed by positively charged residues that form a hairpin-like loop and mimic the matrix targeting sequence (63). N-anchored IMM proteins are also known to possess C-terminal cleavable targeting signals that are processed by IMM-anchored proteases (118, 121).

### TOM–TIM22 pathway for carrier proteins

The TIM22 complex facilitates the import of mitochondrial carrier protein precursors with a noncleavable signal sequence into the IMM (Figs. 4 and 5*B*). Unlike the TOM–TIM23 interaction, TOM–TIM22 interactions are mediated through IMS chaperones (2, 96). Tom70 is the receptor for the carrier family of proteins. In yeast, these proteins are delivered to Tom70 by Hsp70 and Hsp90 (31). After binding and recognition by Tom70, the preproteins are transferred to Tom20–Tom22, before entering the Tom40 pore (30, 44, 122, 123). The TOM complex recognizes the hydrophobic internal targeting sequence. After traversing the Tom40 pore, the preprotein is handed over to the hexameric IMS chaperones Tim9–Tim10 (Fig. 5*B*). Next, Tim12 (of the TIM22 complex) additionally forms a complex with Tim9–Tim10 by displacing one of the copies of Tim10 from Tim9–Tim10 (3, 54, 103). The chaperone complex additionally interacts with Tim54 of the TIM22 complex to complete transfer of the preprotein to the Tim22 channel of the TIM22 complex (Fig. 5*B*) (54, 102, 124, 125, 126, 127, 128, 129, 130). Tim23, which is the core translocase channel of the TIM23 complex, is imported by TIM22 with the assistance of the small Tim8–Tim13 chaperones (131, 132, 133, 134).

### TOM–SAM pathway for OMM β-barrel proteins

The OMM is enriched with multiple copies of transmembrane β-barrels (mitochondrial outer membrane proteins, mOMPs). These are primarily porins (voltage-dependent anion channels in humans), Tom40 (the core channel of the TOM complex), Sam50 (the core protein of the SAM complex), and Mdm10 (found in yeast) (8, 9, 22, 79, 86, 88, 135). mOMP import is therefore a crucial function of the TOM complex (Fig. 4).

The vital components for the import and assembly of mOMPs are the TOM complex, IMS small Tim chaperones (holdases), and the SAM complex (2, 5, 8, 9, 79, 82, 83, 88, 133, 134, 136, 137, 138, 139, 140, 141, 142, 143). All β-barrel precursors carry a noncleavable amphipathic internal targeting signal sequence located in the last β-hairpin of the mOMP (79, 143, 144, 145, 146). This noncleavable signal usually contains the consensus sequence PxGhxHxH (P: polar; G: Gly; h: hydrophobic aliphatic; H: hydrophobic aromatic) (88, 146, 147) and relates evolutionarily to the bacterial β-barrel proteins (8, 9, 145). The import process begins with the interaction of the β-barrel polypeptide with the Tom70 and Tom22 receptors (Fig. 4), followed by translocation through the hydrophobic patch of the Tom40 channel (83, 143, 148). As the polypeptide enters the IMS, the small Tim chaperones Tim9–Tim10 bind and transport the protein to the SAM complex (56, 80, 133, 134, 149). These small Tims act as holdases, preventing the premature folding of the nascent β-barrel (Fig. 4) (134). Insertion of the nascent protein begins in the hydrophilic core of the SAM complex by the formation of a hybrid β-barrel (see Fig. 2), followed by the coordinated folding and release of the β-barrel in the OMM (2, 6, 8, 9, 56, 77, 82, 88, 135, 137, 138, 139, 144, 146, 150).

### Tom70–MIM association for OMM helices

The OMM also contains lipid-anchored α-helices that fall into three subtypes, namely, the helix-anchored proteins (N-terminal, C-terminal tail-anchored) and polytopic α-helices. The hydrophobic anchoring sequences in these proteins are both essential and sufficient for recognition and sorting (151). Tail-anchored α-helices use one of the TOM components (not Tom40) and MIM (10, 12), for insertion in the OMM (Fig. 4) (139, 152). Direct insertion by the MIM complex has also been observed (12, 81). Tom70 acts as the receptor for polytopic α-helices (72, 153, 154). Both MIM and Tom70 have C-terminal domains that reside in the cytosol, which transiently interact for direct cargo transfer from Tom70 to MIM (11, 12). MIM additionally possesses an N-terminal hydrophilic domain in the IMS (85, 92).

The MIM complex is formed of multiple copies of Mim1 (10, 11, 22, 84, 85, 154) and a single copy of Mim2 (12, 155). When polytopic helical proteins are recognized by the Tom70 receptor, it associates with and transfers the polypeptide to the MIM complex, which plays the role of an insertase (10, 40, 81, 154, 156). The SAM complex may additionally facilitate the insertion of α-helical proteins in the OMM by associating directly with the MIM complex (138, 157). Mim1 also plays a role in TOM–CC biogenesis, as it is required for SAM–Tom40 complex formation and Tom5–SAM association (12). Additional mechanistic details behind Tom70–MIM communication, and the precise signal sequence of OMM helices that is recognized by this complex, are yet to be deduced (3, 5, 9, 103, 142, 158).

### Pathway for Cys-rich proteins

**Figure 6:**
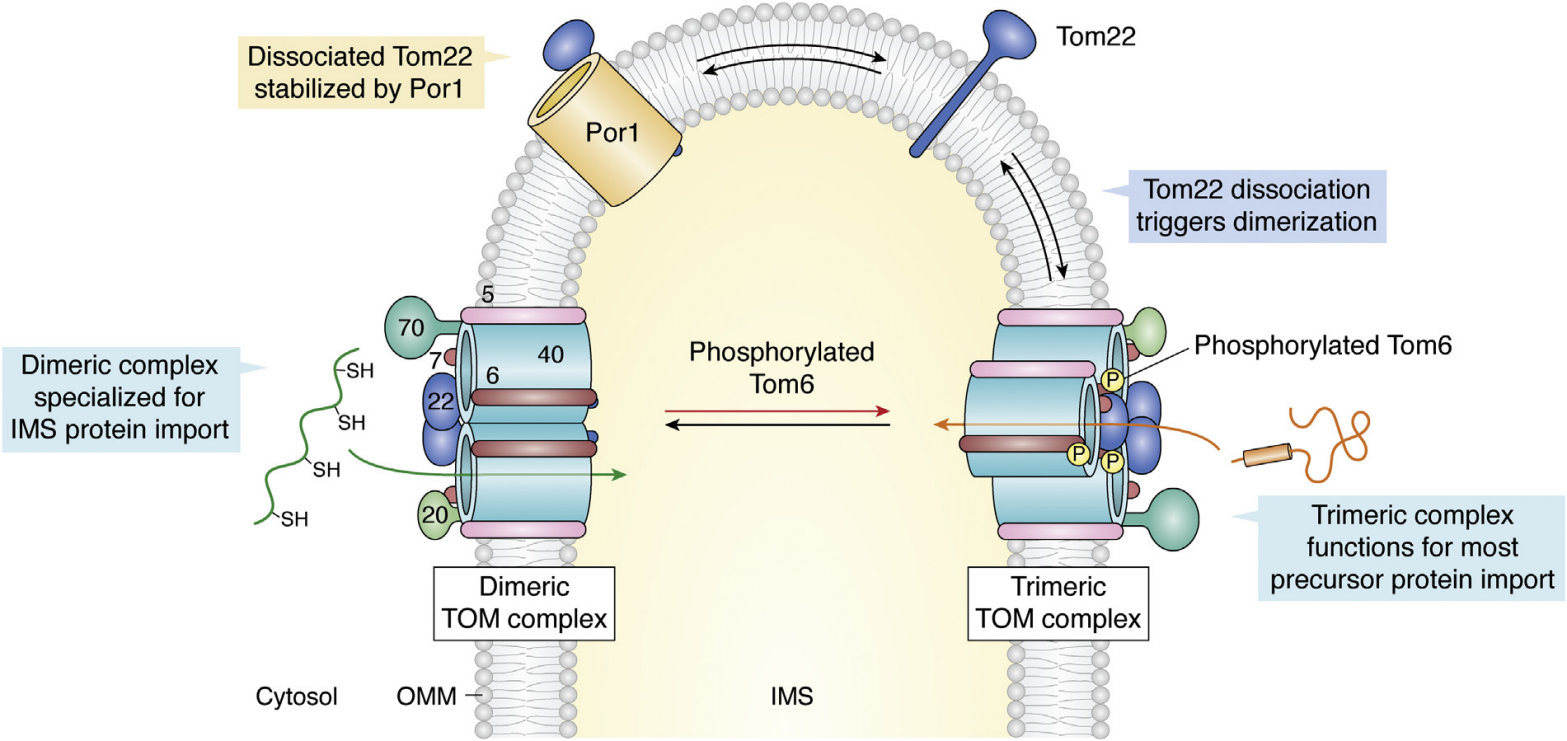
**Dynamic interconversion of the TOM complex for selective substrate import.** Photo-activated crosslinking revealed a dynamic interconversion of the dimeric and trimeric states of the TOM–CC for import of Cys-rich proteins into the IMS and precursors through Tim50–TIM23, respectively. Mitochondrial porin binds and sequesters Tom22, triggering dissociation of the trimeric state. Phosphorylation of Tom6 favors the trimeric assembly. Figure inspired from (50). IMS, intermembrane space; TIM, translocase of the inner mitochondrial membrane; TOM, translocase of the outer mitochondrial membrane; TOM–CC, TOM core complex.

Several of the ∼50 yeast and ∼130 mammalian proteins destined for the IMS (including the small Tim chaperones; (54, 127)) are of low molecular weight (7–25 kDa) and carry an internal targeting signal sequence, distinct Cys-rich motifs, and are stabilized by structural disulfide bonds (107, 159, 160, 161, 162, 163, 164). An internal amphipathic helix formed by cysteines and its proximal residues is sufficient for IMS targeting (63, 130, 161, 163, 165). These preproteins are imported through Tom40 in their reduced unfolded state (Fig. 4) and captured first by the small Tim chaperone Tim9–Tim10 in the IMS (14, 126, 128, 129, 133, 159, 160, 161). This import requires a dimeric TOM complex (Fig. 6) (50). The process is completed by Mia40, an IMM-anchored oxidoreductase, which is a subunit of the mitochondrial intermembrane space assembly (MIA) machinery (166, 167, 168, 169, 170, 171). Mia40 serves as the receptor for Cys-rich preproteins with an internal hydrophobic signal sequence and facilitates release of the mature protein in the IMS (Fig. 4) (163, 164, 165, 166, 170).

Recent studies show that the TOM complex undergoes dynamic interconversion between dimeric and trimeric states for the selective import of Cys-rich and preprotein sequences, respectively (Fig. 6) (50). The mitochondrial porin (Por1 in yeast) sequesters Tom22, triggering the conversion of trimeric TOM to its dimeric state, which becomes import-competent for Cys-rich proteins by association with Mia40. Mia40 interacts with the preprotein through its hydrophobic pocket and formation of transient disulfide bonds (107, 161, 164). Mia40 plays the role as disulfide carrier (170, 171), while the other protein in the MIA complex, Erv1 (Essential for respiration and viability 1), generates disulfide bonds in the substrate (172, 173, 174, 175). The receptor function of Mia40 ensures that the mature protein is released specifically into the IMS.

### Pathway for other IMS proteins

A few IMS-targeted larger (multidomain) proteins lack the Cys-rich motifs and are imported through a bipartite mechanism (5, 77). These proteins possess cleavable N-terminal matrix-targeting sequence that is believed to be recognized by Tom70 and is followed by import through Tom40 and transfer to TIM23 in the IMM. Before the import is completed, the stop transfer sequence is recognized, and inner membrane peptidases cleave and release the C-terminal multidomain region into the IMS (176). Several apoptosis-associated proteins, cytochrome *b*_2_ and cytochrome *c* peroxidase, are imported through this pathway (177, 178, 179, 180, 181).

### Interplay of Tom40 and endoplasmic reticulum membrane proteins

Largely, mitochondria function independently during the import, targeting, and sorting of the nuclear-encoded mitochondrial proteins. One interesting exception that has been identified is the cytosolic protein NDUFS4 (NADH:ubiquinone oxidoreductase subunit S4), which forms a component of complex I of the electron transport chain (182). Its translocation to IMM requires interaction with Bap31 (B-cell receptor-associated protein 31), which is an endoplasmic reticulum protein (183). The interaction of Bap31 and Tom40 establishes mitochondria–endoplasmic reticulum cross-talk. This process also regulates mitochondrial homeostasis by controlling the mitochondrial localization of NDUFS4 and NDUFB11 (NADH:ubiquinone oxidoreductase subunit B11) for interaction with and import by Tom40 (184).

## TOM dysregulation and neurodegeneration

Mitochondrial dysfunction, which often relates to import-associated defects (185), leads to several neurodegenerative and age-related disorders. Mitochondria play a pivotal role in the pathogenesis of Huntington's disease, Parkinson's disease, and Alzheimer’s disease (158, 186, 187, 188, 189). Nearly all diseases arising from mutagenesis or gene polymorphism have been identified for the inner membrane complexes. However, heterozygous mutations in the TOM complex constituents have also been identified, which impair protein import, whereas loss-of-function alleles directly cause embryonic lethality (188). Misimport of cytosolic proteins that lead to mitochondrial dysfunction, which are more closely associated with the TOM complex and mOMPs, particularly affect neuronal development, neurotransmission, calcium flux, synaptic contacts, and culminate in neurodegenerative diseases. The known pathological consequences of misimport defects associated with the TOM complex are listed in Table 1 (187). Molecular events in each disease are still being deduced, and studies that discuss the probable association of the TOM complex with various cancers are reviewed elsewhere (158, 187, 188, 189).

**Table 1:** Pathological consequences of TOM import defects^a^

Pathology	Import defect(s)	Known consequence(s)	Model organism/system
Alzheimer’s disease (AD)	APP accumulation in Tom40 and Tim23 channels, with higher levels in AD susceptible brain regions.	Inhibition of import of respiratory complex IV (CIV) 4 and 5b and subsequent reduction in CIV activity, leading to increased ROS.	Human AD brains.
	Chronic, sublethal Aβ exposure induces a significant reduction in mitochondrial protein import.	Reduction in Δψ, altered mitochondrial morphology, and increased ROS production.	PC12 cells.
	Tau accumulation in OMM and IMS and interactions between N-terminal Tau fragment with OPA1 and Mfn1.	N/A	HEK293T cells, HeLa cells.
Parkinson’s disease (PD)	α-Syn localizes to and accumulates within mitochondria, mediated by a cryptic noncanonical MTS, in an ATP- and Δψ-dependent manner	N/A	Human dopaminergic neuronal cultures, PD brains.
	A53T version of α-syn is imported more efficiently than WT variant.	May account for faster development of cellular abnormalities seen in cells expressing the A53T version of α-syn compared to the WT.	Human dopaminergic neuronal cultures, PD brains, A53T mutant α-syn–inducible PC12 cell lines.
	Mitochondrial α-syn accumulates at IMM and interacts with respiratory complex I (CI).	Reduction in CI activity, increase in ROS production, inducing oxidative stress.	Human dopaminergic neuronal cultures, PD brains, rat *SN* neurons, human neuroblastoma cell line (SK-N-MC cells).
	S129 phosphorylated α-syn binds tightly to Tom20, inducing loss in Tom20–Tom22 interaction.	Impaired protein import, loss of Δψ, reduced respiratory capacity, and increased oxidative stress. Rescued by *in vivo* knockdown of endogenous α-syn and by *in vitro* Tom20 overexpression.	SH-SY5Y cells and dopaminergic neurons from *SN* of postmortem PD patient brains.
	Tom40 downregulation, corresponding with α-syn accumulation in PD brains.	N/A	Midbrain of PD patients and α-syn transgenic mice.
	Excessively low levels of mitochondrial import in cells from *PINK1*- and *PARK2*-linked PD patients.	N/A Import defects reversed by phosphomimetic ubiquitin in cells with residual Parkin activity.	Cells from *PINK1*- and *PARK2*-linked PD patients.
Huntington’s disease (HD)	Disease variant HTT localizes to mitochondria and directly interacts with the TIM23 complex.	Inhibited import and subsequent respiratory dysfunction, triggering cell death, rescued by TIM23 overexpression.	Isolated mitochondria from human HD brains, primary neurons expressing HTT variant, forebrain synaptosomal mitochondria in HD mice at early stages of HD.
	Dysfunctions in MIA pathway associated with mutant HTT: reduced levels and ratio of Erv1 and Mia40.	Reduced import of MIA pathway precursors, CIV assembly defects, deficient respiration, alterations in mtDNA, altered mitochondrial morphology.	Neuronal cell lines.

Abbreviations: α-syn, α-synuclein; APP, amyloid precursor protein; HTT, Huntingtin gene; Mfn1, mitofusin-1; mtDNA, mitochondrial DNA; MTS, mitochondrial targeting signal; OPA1, optic atrophy type 1; PARK2, parkin RBR E3 ubiquitin protein ligase; PINK1, PTEN-induced putative kinase; ROS, reactive oxygen species.

a Contents obtained from and reproduced with permission from Needs *et al.* (2021) (187).

## Unanswered questions and future perspectives

The availability of detailed structural information on TOM complex organization, coupled with functional studies in model organisms and the mapping of genetic mutations causing a defective TOM interactome, have together augmented our understanding of how this essential complex maintains mitostasis in all cells. These findings from the last 3 decades now provides the foundation to address questions on the evolution of the TOM complex, particularly the unique 19-stranded β-barrel structure of Tom40. Unanswered questions remain on the stepwise assembly of Tom40 and the molecular mechanisms that regulate the folding of this vital core protein. Combinatorial studies involving biophysical methods and biochemical approaches paired with *in vivo* biology will resolve how structural changes in Tom40 regulate the TOM complex, and whether substrate-induced changes in the TOM complex dynamics affect its import efficacy. The role of the mitochondrial outer membrane (including cardiolipin, cholesterol, and phosphatidylinositol) in regulating this complex is yet to be deduced. Furthermore, fundamental studies mapping residues vital for the folding and function of Tom40 (with translational studies to human TOMM40) will provide atomic insight on TOM-associated diseases in humans. Interestingly, the *in vivo* importance of the gating characteristic of Tom40 (observed *in vitro*) is yet to be understood. The mapping of other regulators in the cytosol and IMS will provide a wider impact on our understanding of the interdependence of mitochondrial biogenesis and bioenergetics with other cellular compartments in particular, and the cell cycle as a whole.

## Conflict of interest

The authors declare that they no conflict of interest with the contents of this article.
